# Quantification and modeling of mechanical degradation in lithium-ion batteries based on nanoscale imaging

**DOI:** 10.1038/s41467-018-04477-1

**Published:** 2018-06-14

**Authors:** Simon Müller, Patrick Pietsch, Ben-Elias Brandt, Paul Baade, Vincent De Andrade, Francesco De Carlo, Vanessa Wood

**Affiliations:** 1 Department of Information Technology and Electrical Engineering, ETH, Zurich, 8092 Switzerland; 2 Advanced Photon Source, Argonne National Laboratory, Lemont, 60439 USA

## Abstract

Capacity fade in lithium-ion battery electrodes can result from a degradation mechanism in which the carbon black-binder network detaches from the active material. Here we present two approaches to visualize and quantify this detachment and use the experimental results to develop and validate a model that considers how the active particle size, the viscoelastic parameters of the composite electrode, the adhesion between the active particle and the carbon black-binder domain, and the solid electrolyte interphase growth rate impact detachment and capacity fade. Using carbon-silicon composite electrodes as a model system, we demonstrate X-ray nano-tomography and backscatter scanning electron microscopy with sufficient resolution and contrast to segment the pore space, active particles, and carbon black-binder domain and quantify delamination as a function of cycle number. The validated model is further used to discuss how detachment and capacity fade in high-capacity materials can be minimized through materials engineering.

## Abstract (web summary)

Silicon is a promising electrode material for lithium-ion batteries; however, morphological changes shorten battery lifetimes. Here the authors use imaging techniques based on electrons and X-rays to quantify such processes at micro- and nanoscales and suggest routes to mitigate battery degradation.

## Introduction

Applications are demanding more from lithium-ion batteries (LIBs): higher energy and power densities, improved safety, longer cycle life, and lower cost^1–3^. To prolong cycle life, it is important to gain deeper understanding of the degradation mechanisms in high-capacity LIB electrodes.

In LIB electrodes, micrometer-sized electrochemically active particles are mechanically stabilized by a polymeric binder. With the exception of graphitic anodes where the active particles are sufficiently conductive, most electrodes contain an electrically conductive additive, such as a nanoscale carbon black, that is dispersed with the binder^4–6^. The carbon black-binder domain can be thought of as a network that mechanically and electrically connects the active material particles^7^. For an active particle to remain electrochemically active and contribute to the capacity of the electrode^8^, the carbon black-binder network and the active particle must remain in contact.

Many high-capacity materials undergo volumetric expansion and contraction or mechanical changes such as fracture during cycling, which can lead to detachment of the active particles from the carbon black-binder network. This is an effect that occurs both in high-voltage cathode materials, such as LiMn_2_O_4_, which rupture during cycling^9^, and in high-capacity anode materials such as silicon that undergo alloying reactions with lithium and large volumetric changes (280% in the case of silicon) upon (de)lithiation^10–12^.

High-capacity graphite-silicon composite electrodes are selected as a model system for studying detachment. In such systems, 5–20 wt.% of silicon is added to graphitic negative electrodes, boosting the theoretical specific capacity of a graphite electrode from 372 Ah/kg to 532 Ah/kg (5 wt.% silicon) or to 1013 Ah/kg (20 wt.% silicon)^8,13–15^. To achieve the goal of mitigating the severe degradation that occurs in pure silicon electrodes, the silicon is embedded into mechanical robust graphite, which undergoes small volumetric changes on the order of 10% upon (de)lithiation^16^ and exhibits excellent cycling stability under normal operating conditions^17,18^.

**Fig. 1:**
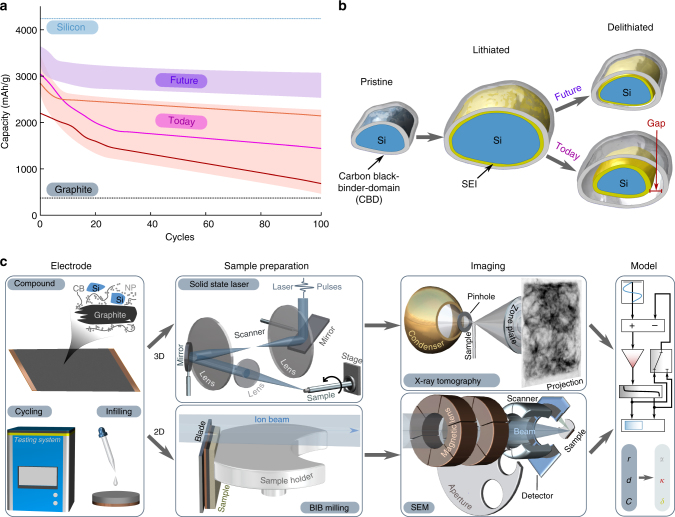
Motivation and methods. **a** Comparison of the specific charge capacity of graphite (gray line) and silicon (light blue line) electrodes. The red shaded area shows example performances of today’s graphite-silicon compound electrodes leaving space for improvement in the future (purple shaded area). **b** Illustration of a major degradation mechanism in silicon electrodes. Upon lithiation of silicon, a solid electrolyte interphase (SEI) film (yellow) forms on the silicon particle surface and the carbon black-binder domain (light gray) is pushed outwards. Upon delithiation, the silicon particle shrinks, thereby detaching from the carbon black-binder domain. The arising gap (red) electrically isolates the silicon particle, thereby leading to rapid capacity fade. **c** Schematic of experimental steps and methods used in this work. From left to right: silicon-graphite mixed electrode sheets are fabricated, electrochemically cycled, and infiltrated with epoxy resins. For 3D transmission X-ray tomographic microscopy imaging the infiltrated samples are milled to the required size using a pulsed solid-state laser, while for 2D scanning electron microscopy imaging cross-sections of the electrodes are prepared using Broad Ion Beam milling. The combined 2D and 3D data are quantitatively analyzed and used to develop a predictive model that links the mechanical properties of the composite electrode ($\alpha ,\kappa$) and SEI growth rate ($\delta$) for a particle of radius $r$ to the detachment distance $d$ and the capacity fade $C$

Graphite-silicon composite electrodes are an excellent model system to study detachment since the capacity fade observed in these electrodes (Fig. 1a) is attributed in large part to the mechanical detachment of the carbon black-binder domain from the silicon particles^19,20^. Additionally, while graphite particles form a thin and stable solid electrolyte interphase (SEI) after only few cycles^21^, the SEI on silicon continuously grows and consumes lithium ions even after prolonged cycling. As silicon particles fracture due to the large volumetric expansion, fresh silicon surfaces are exposed to the electrolyte, initiating additional SEI formation^22^ (Fig. 1b).

In this work, the capacity loss associated with the loss of electrical contact between the active materials and the rest of the composite electrode due to delamination of the carbon black-binder network is studied. Procedures are developed for visualizing and quantifying the detachment using X-ray and electron microscopy. The weak contrast among graphite, the carbon black-binder domain, and the SEI layer on the silicon particles presents a particular challenge for imaging and quantification, which suggests that these developed methods could be widely applicable. Furthermore, the experimental measurements of detachment and the electrochemical data are used to develop and validate a model that describes the detachment of active particles from the carbon black-binder network as a result of electrochemical cycling. The model provides insights into how LIB life can be improved by rational electrode design, such as selection of active particle size or binders.

## Results

### Nanoscale imaging and multi-phase data segmentation

Quantification of detachment requires five-phase segmentation. Pore space, silicon particles, graphite particles, carbon black-binder domain, and, for cycled samples, the SEI layer must be reliably differentiated.

To date, few studies have successfully imaged the carbon black-binder domain and, to the best of our knowledge, none have shown its detachment from the active particles. Previously the carbon black-binder domain has been investigated in contrast-rich cathode environments^23^ and polyethylene-oxide binders have been stained with lithium iodide^24^. Other studies have statistically modeled the carbon black-binder domain^25^ or applied correlative tomography approaches^26^.

Indeed, imaging the carbon black-binder domain presents major challenges. First, imaging the nanometer sized features of the carbon black-binder domain requires techniques with very high-spatial resolution. Second, in both attenuation-based X-ray and electron backscatter imaging, the signal collected from the sample increases with the core charge number of its elements^27,28^.

This means that the signal collected from the light elements contained in the carbon black-binder domain will be weak. Moreover, the signal will be of similar magnitude to that collected from the graphite particles, which will result in weak image contrast.

To overcome these difficulties, we replace 50 vol.% of the carbon black with nanoparticles of similar size and shape (Supplementary Note 1). While Harris and colleagues (unpublished observations) used iron nanoparticles, we choose carbon-coated copper nanoparticles, because copper is (i) a heavy element that provides contrast in both imaging techniques, (ii) a good electrical conductor as carbon black^29^, and (iii) stable under electrochemical operation conditions in a negative electrode. Indeed, (partially) replacing carbon black with the nanoparticles does not alter the electrochemical performance (see Supplementary Fig. 1b).

Samples for imaging are selected from pristine electrodes and electrodes that have been fully delithiated after electrochemical operation for one, three and ten cycles in half-cell configuration (Supplementary Note 1).

As illustrated in Fig. 1c, to obtain detailed three-dimensional electrode microstructures, we perform attenuation-based transmission X-ray tomographic microscopy (TXTM) experiments.

To ensure mechanical rigidity, the electrode samples are vacuum infiltrated with epoxy resin (Supplementary Note 2). A fast laser-milling based technique^30^ is implemented to obtain 50-µm diameter samples, which are necessary to operate the TXTM in non-local tomography mode with a voxel size of ~30 nm (Supplementary Note 3).

**Fig. 2:**
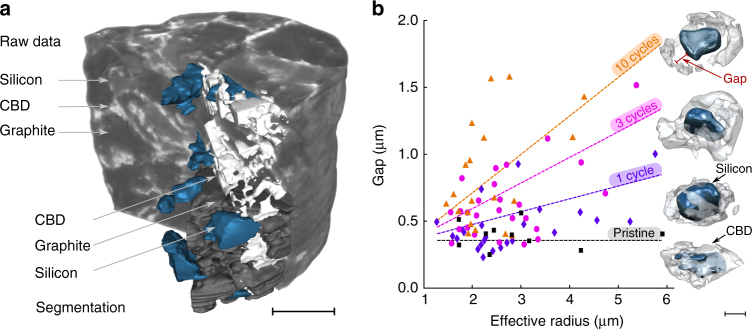
Transmission X-ray tomographic microscopy. **a** Tomographic raw data of a pristine silicon-graphite electrode shown with multi-phase segmentation, whereby the silicon particles (blue), the graphite particles (dark gray), the carbon black-binder domain (light gray), and the pore space can be distinguished. Scale bar: 15 µm. **b** Average gap between the surface of a silicon particle and the corresponding carbon black-binder domain as a function of the respective particle effective radius. The data have been acquired in electrodes in the pristine state (black squares) and in fully delithiated electrodes after one cycle (purple diamonds), after three cycles (magenta circles), and after ten cycles (orange triangles). Gaps increase with increasing cycle number and increasing silicon effective particle radius, which is confirmed by renderings of individual particles extracted from the segmented tomographic data. The dotted lines are included to guide the eye. Scale bar: 5 μm

Figure 2a shows the reconstructed tomogram of a pristine sample. The stained carbon black and silicon particles can be distinguished, and using a priori knowledge about particle size and porosity (Supplementary Note 5), it is further possible to distinguish the weakly attenuating graphite particles from the pore space, such that four-phase segmentation is achieved: the silicon particles, the graphite particles, the carbon black-binder domain, and the pore space.

Since the sample volume contains only 10–20 silicon particles, scanning electron microscopy (SEM) imaging is employed to enlarge the data pool (Fig. 1c). Here an imaging area 40 × 50 μm is selected, which leads to pixels with edge lengths of 21 nm. Quantitative backscatter SEM imaging requires planar sample surfaces to avoid scattering signals from deeper within the pore space^31^. To the best of our knowledge, it is shown for the first time that triphenyl bismuth^32,33^ stained epoxy can be vacuum infiltrated in microstructures and allows for tunable radiopacity and/or SEM backscatter contrast (Supplementary Note 4). Broad-ion-beam (BIB) milling of the cross-sections leads to flat and polished surfaces^34^. Chromium is sputter-coated on top of the sample to prevent local charging effects^35^.

### Quantifying carbon black-binder domain detachment

The goal of the imaging experiment is to quantify the detachment as a function of electrochemical cycle number and silicon particle size in order to develop the model.

First, with the segmented tomography data, the average distance between each silicon particle and the nearest carbon black-binder domain is evaluated based on a ray tracing approach (Supplementary Note 7). In Fig. 2b, the averaged distances are plotted for every silicon particle as a function of the radius of the particle for electrodes that are uncycled, cycled once, cycled three times, and cycled ten times. The gap distance between the silicon surface and the carbon black-binder domain increases with growing particle radius and progressing cycle number, as indicated by the dotted lines, which are included as a guide to the eye.

**Fig. 3:**
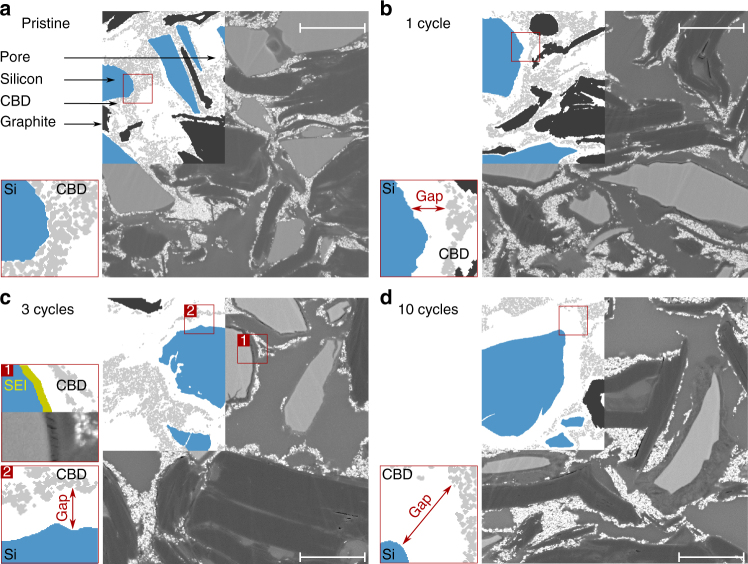
Scanning electron microscopy. Cross-sectional backscatter scanning electron microscopy images of epoxy infiltrated electrodes in combination with the corresponding multi-phase segmentation for **a** a pristine electrode and fully delithiated electrodes after **b** one cycle, **c** three cycles, and **d** ten cycles. The red boxes show magnifications at silicon particle surfaces, highlighting the formation and growth of the gap between the silicon particles and the carbon black-binder domain (CBD). The additional inset in **c** shows that the space between the silicon particles and the carbon black-binder domain is not completely filled with epoxy. Instead, the weakly backscattering dark layer in the vicinity of the silicon particle points to the formation of a thick solid electrolyte interphase (SEI) layer. Scale bars: 5 μm

As explained above, to obtain more statistics, the 2D SEM cross-sections are analyzed (Supplementary Note 6 and 8). The SEM images in Fig. 3 depict the detachment process. In the upper left corner of each gray scale image, color is used to highlight the different phases. A zoomed-in image of a region (indicated with the red box) is shown in the lower left. In the pristine state (Fig. 3a), the silicon particles (blue) are surrounded by the carbon black-binder domain (light gray). After the first cycle (Fig. 3b), a gap between the silicon particles and the carbon black-binder domain opens up. This gap increases with cycling, and, after three cycles (Fig. 3c), the first cracks in the silicon particles emerge and the SEI layer (yellow) becomes visible^36^. After ten cycles (Fig. 3d), the carbon black-binder domain is almost fully detached from all silicon particles, while it remains in contact with the graphite particles.

**Fig. 4:**
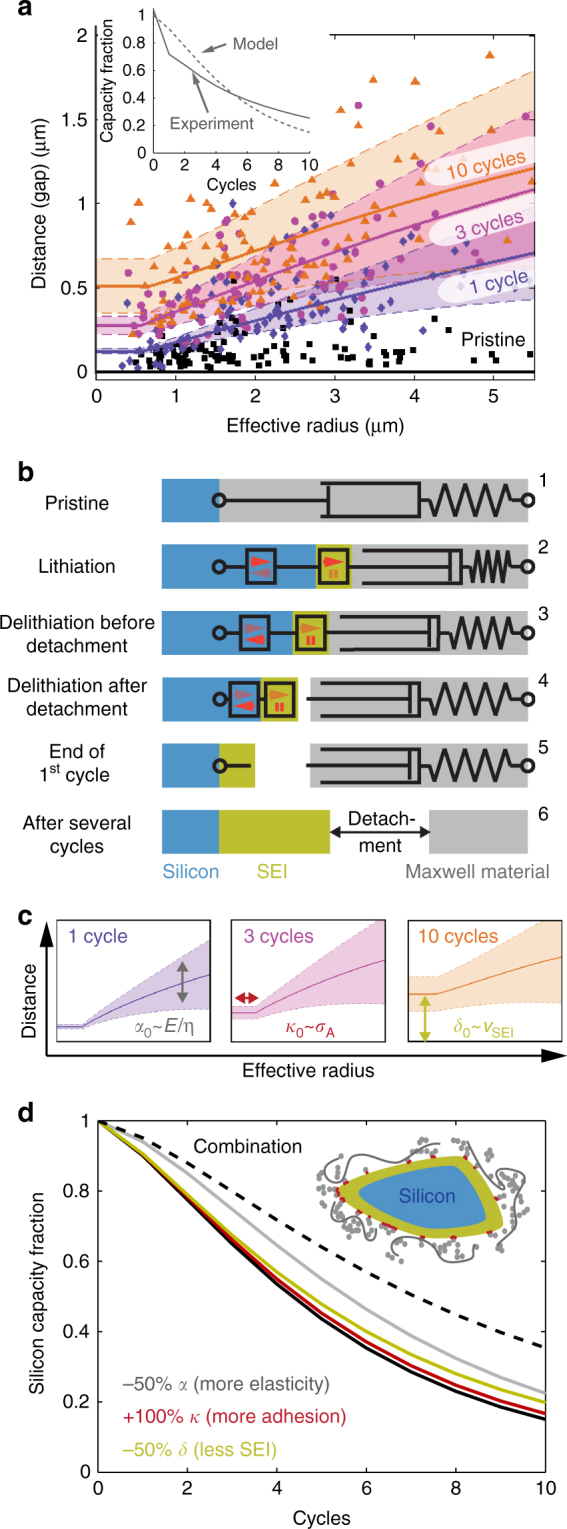
Quantitative analysis. **a** Transmission X-ray tomographic microscopy data from Fig. 2b superimposed with additional data obtained from stereographic conversion of the 2D scanning electron microscopy data presented in Fig. 3 for pristine electrodes (black square), and electrodes cycled once (purple diamonds), three times (magenta circles), and ten times (orange triangles). The colored solid lines and shaded regions show the distance expectation values and corresponding standard deviations according to the model presented in the main text. The inset compares the silicon capacity retention of the experimental data (solid line) with the simulation of the model (dashed line). **b** Sketch of the fundamental components of the model and operation at various stages in the electrochemical cycling process. Silicon expansion and contraction, as well as solid electrolyte interphase (SEI) growth are represented by linear actuators, while the carbon black-binder domain and the electrode material in the direct vicinity of a silicon particle are represented as Maxwell material that consists of a spring element with a damper element in series. **c** Illustrations of the three parameters $\alpha _0 \propto E{\mathrm{/}}\eta$, $\kappa _0 \propto \sigma _{\mathrm{A}}$ and $\delta _0 \propto v_{{\mathrm SEI}}$ that affect different parts of the model. **d** Simulation of silicon capacity retention for different fictive parameter configurations, gray line: −50% α (more elasticity), red line: +100% κ (more adhesion), green line: −50% *δ* (less solid electrolyte interphase)

Performing a stereographic conversion^37^ of the particle size and gap measurements from the SEM images (Supplementary Note 9), enables it to be plotted with the 3D TXTM data (Fig. 4a).

The large deviations in gap size that are observed even for similar-sized particles in the same electrode arise from local microstructural inhomogeneities around the silicon particles (i.e., binding), which lead to a statistically distributed number of active cycles that each individual particle undergoes until it irreversibly detaches from its environment and stops participating in the electrochemical reactions.

### Modeling the detachment of the carbon black-binder domain

In order to understand how to systematically improve the cycle life of high-capacity electrodes through materials engineering, a model is developed to quantitatively describe the detachment process.

It has been reported that the major mechanisms that lead to electrochemical degradation in silicon-based electrodes are^38^ (i) the irreversible and continuous consumption of cyclable lithium in the SEI formation processes and other side reactions, (ii) the (kinetic) trapping of lithium in the active materials^39^, and (iii) the (partial) electric isolation of active material from the environment or equivalently rising electric SEI and contact resistances^40,41^. Mechanism (i) can be excluded as a cause of degradation for the electrochemical experiments reported here since the metallic lithium counter electrode provides a quasi-infinite reservoir of lithium ions. By adding a potentiostatic step at 1.5 V after the galvanostatic cycling process, it was shown that the amount of lithium that is (kinetically) trapped in the silicon active material is rather small (ca. 15%, Supplementary Fig. 12). While other routes of degradation cannot be completely ruled out, the above reasoning together with the clear observation of silicon particle detachment (Figs. 2b and 3) and that the electrodes containing only graphite as the active material show no signs of degradation (Supplementary Note 1), suggest that (electric) isolation of the silicon particles is the dominant degradation mechanism at play. Our model therefore directly links the observed detachment distances with the rate of electrochemical degradation.

We hypothesize that the likelihood for a given active particle to remain electrically connected to the electrode network (and thus to contribute to the electrode’s charge capacity) decreases with increasing average distance *d* between the silicon particle surface and its environment (Supplementary Note 7 and 8). We assume a linear ‘disconnection probability’, i.e. $p\left( d \right) = \zeta _0d$ in each cycle, where the slope $\zeta _0$ is a variable parameter which describes how much the probability to disconnect increases for a given increase in *d*.

The local environment of the silicon particles (consisting of the carbon black-binder domain, graphite particles, other silicon particles, and pore space) is treated as an effective medium^42,43^. This effective medium is modeled as a Maxwell material^44^, which is the serial connection of a dissipative damper element (with viscosity $\eta$) and a spring element (elastic modulus $E$) (Fig. 4b). For the purpose of this study $E$ and $\eta$ can be lumped into a single-material parameter $\alpha _0 \propto \frac{E}{\eta }$ (Supplementary Note 10).

We further assume that detachment occurs during the delithiation process as silicon particles shrink, if the tensile stress between the particle surface and the Maxwell material exceeds the maximal adhesion of the binder^45^, which is represented by $\kappa _0$ (see Supplementary Note 10).

Finally, besides the repeated volumetric changes of the silicon particles, the formation of a SEI layer on the silicon surface causes further mechanical deformation. Here, $\delta _0$ represents the SEI thickness growth rate, which is assumed to be constant over time during the lithiation processes^21^ (Supplementary Note 10).

The operational aspects of the model are illustrated schematically in Fig. 4b. In the pristine state of the electrode, the Maxwell material is connected to the silicon particle surface (sketch 1). Upon lithiation, the expansion of the silicon particle in combination with SEI growth compresses the Maxwell material (sketch 2). Because of its damping property, the compression is only partially reversible. This means that the Maxwell material can detach from the SEI surface upon delithiation, if the critical adhesion surface stress is exceeded (sketches 3 and 4). By the end of the first electrochemical cycle, a gap opens between the SEI on the silicon particle and the remainder of the electrode. This gap gradually increases in subsequent cycles (sketches 5 and 6). The detailed mathematical description of our model is provided in Supplementary Note 10.

The model is assessed by comparing its predictions for the detachment distance (solid lines and color shaded regions in Fig. 4a) and the rate of electrochemical degradation (dashed line in the inset of Fig. 4a) to the corresponding experimental data using an optimal set of the four fit parameters $\alpha _0$, $\kappa _0$, $\delta _0$, and $\zeta _0$. The color shaded regions take into account the standard deviations of the aforementioned expected distribution of gaps due to microstructural inhomogeneity and the solid lines represent their means.

All four parameters define distinct features of the model predictions (Fig. 4c). $\alpha _0$ controls the shape and slope of the lines in the large particle limit. The binder adhesion $\kappa _0$ defines the particle size below which tensile stresses are too low to cause detachment (flat plateau in the small particle limit). In this regime, the only contribution to the gap is the SEI thickness, which is determined by $\delta _0$ for a given number of cycles. Finally, $\zeta _0$ links the gap distance to electrochemical degradation.

### Model validation

In order to validate our model, the reasonable agreement of the obtained fit parameters ($\alpha _0 \approx {{0.83}_{ - 0.09}^{ + 0.23}}$,$\kappa _0 \approx {{0.02}_{ - 0.02}^{ + 0.03}}\,{\mathrm{\mu}} {\mathrm{m}}$, $\delta _0 \approx {{0.12}_{ - 0.05}^{ + 0.02}}\,{\mathrm{\mu}} {\mathrm{m}}$, and $\zeta _0 \approx {{0.163}_{ - 0.005}^{ + 0.006}}\,{\mathrm{\mu}} {\mathrm{m}}^{ - 1}$) with literature values were assessed. Note that bootstrapping was used to assess the above reported uncertainty ranges of the fit parameters (Supplementary Note 11).

The parameter *α*_0_ translates into a ratio of the effective elastic modulus and the effective electrode viscosity $\frac{E}{\eta} \approx 0.04_{ - 0.005}^{ + 0.01}\,{\mathrm{h}}^{ - 1}$. While effective elastic moduli of $E = 137 - 440\,{\mathrm{MPa}}$ have been measured for compressed silicon electrodes fabricated with different binders^46^, no effective electrode viscosity measurements exist. Based on complementary measurements (Supplementary Note 11), an effective electrode viscosity of ${\eta} \approx 1000\,{\mathrm{GPa}}\,{\mathrm{s}}$ (similar to glucose at room temperature) is found, which translates into a ratio $\frac{E}{\eta } \approx 0.5 - 1.6\,{\mathrm{h}}^{ - 1}$. This deviation is reasonable, since viscosities of complex and rather stiff materials, such as tarmac or glasses, can span a range of several orders of magnitude, depending on preparation and measurement conditions^47,48^. It is also noted that while our model probes the ratio ${\frac{E}{\eta }}$ microscopically in the neighborhood of individual silicon particles, the effective elastic moduli and our guess for the viscosity comes from macroscopic measurements.

While a precise estimate for $\kappa _0$ cannot be obtained due to the lack of (i) more and (ii) more precise image data at the small particle end, we are able to provide an upper bound of $\kappa_0$<0.05 μm (Supplementary Note 11). To provide an upper bound, particle sizes are required to avoid detachment stemming from the repeated volumetric changes of silicon, which is in qualitative agreement with recent trends to nano-size silicon for LIB applications to avoid particle cracking^8,11,38^.

The value for $\delta _0$ translates into an average SEI growth rate of 6 nm h^−1^, which is in good agreement with literature, where the growth of thick SEI layers has been observed on silicon surfaces at effective rates in the range 1–25 nm h^−^^1^ ^49–52^.

Based on this, we conclude that our model captures the key elements of binder detachment processes in LIB electrodes.

## Discussion

To better understand the effect of the elasto-mechanical properties, the adhesion, and the SEI growth rate on the electrochemistry, our model is used to simulate the fade of active silicon capacity for different parameter combinations (Fig. 4d).

If the composite electrodes are more elastic and less dissipative (i.e., smaller $\alpha _0 \propto \frac{E}{\eta }$), the cyclic compressions become more reversible such that the gap sizes decrease. Similarly, a slower SEI growth (smaller $\delta _0$) and higher adhesion (larger$\kappa _0$) improve the electrochemistry by decreasing the gap size.

According to the model, an improvement of all three parameters by a factor of two would increase the available silicon capacity fraction after 10 cycles from ~20% to almost 40%. While this quantitative result significantly depends on the assumed shape of the ‘drop-out’ probability distribution $p(d)$, the model gives qualitative directives for future work on mitigation of the delamination of the carbon black-binder domain and the accompanying capacity fade.

Indeed, it has been reported that silicon-based electrodes perform poorly with rather stiff polyvinylidene difluoride based binders^53^, while improved cycling stability was found for more elastic binders (improvement of $\alpha _0$)^46^. Furthermore, improved binder adhesion ($\kappa _0$)^54^, and electrolytes with improved voltage stability windows or SEI stabilizing additives (improvement of $\delta _0$)^55^ benefit cycling performance. The question of optimal silicon particle size remains challenging: while smaller silicon particles undergo smaller absolute volumetric changes and are therefore less likely to detach from the environment, the higher specific surface area shortens LIB longevity due to accelerated side reactions and loss of usable lithium in the continuous SEI formation processes.^38,56^ Composite active materials with embedded nano-sized silicon may therefore be a promising way to solve this challenge.

In summary, the simple 1D model developed and validated here provides basic insights into how the active particle size, the viscoelastic parameters of the composite electrode, the adhesion between the active particle and the carbon black-binder domain, and the solid electrolyte interphase growth rate impact detachment and capacity fade. It can be used to make rational choices about active particle size, binder selection, and the use of electrolyte additives. The procedures for visualizing and quantifying the detachment of the carbon black-binder domain can be used to gain deeper insights into the structure of these networks in porous electrodes and how to optimize them. Indeed recent studies suggest that transport parameters of an electrode microstructure, such as porosity or tortuosity, can be highly affected by the carbon black-binder network^23,25,57^. Optimizing these transport characteristics together with the mechanical properties is a key step for performance of next-generation battery electrodes.

## Methods

### Electrode preparation

Silicon-graphite mixed electrodes with 15 wt.% silicon (BASF, ${\mathrm{SiO}}_{x}$, $x \approx 1$, 2275 mAh g^−1^), 70 wt.% graphite (Hitachi, MagE), 10 wt.% polyvinyldiene difluoride (PVDF) binder (Kynar Flex® HSV900), and 5 wt.% carbon black (Imerys, C65) are prepared. For better imaging contrast, the carbon black in the carbon black-binder domain is (partially) substituted by carbon-coated copper nanoparticles (US Research Nanomaterials Inc.). “Hybrid electrodes” refer to the ones where 50 vol.% of the carbon black is replaced by carbon-coated copper nanoparticles, and “nanoparticle electrodes” refer to ones having all carbon black replaced by nanoparticles. 13-mm disk-shaped electrodes are punched out from the electrode sheets, compressed with 800 kg, and transferred into an Argon-filled glovebox.

### Electrochemical experiment

Coin cells are assembled using lithium metal counter electrodes (Alfa Aesar, lithium foil, 99.9%), 500 μL standard LP50 electrolyte (BASF, 1 M LiPF6 in ethylene carbonate: ethyl methyl carbonate = 1:1 by weight), and 250 μm thick glass fiber separators (Whatman® glass microfiber filter). The cells are operated galvanostatically at a C/20 rate between 10 mV and 1.5 V for 1, 3, 10, and 30 cycles using MPG2 and VMP3 battery cycling systems (Biologic). An additional 20 h potentiostatic step at 3 V is added at the end of each protocol. More detailed information can be found in Supplementary Note 1.

### Transmission X-ray tomographic microscopy sample preparation

The hybrid electrode samples are vacuum infiltrated with epoxy (Buehler, EpoThin2) to ensure mechanical integrity (see Supplementary Note 2). Disks of 1-mm diameter are punched out and glued onto custom-made invar sample holders. The samples are laser milled to a diameter of 50 μm (Supplementary Note 3).

### Transmission X-ray tomographic microscopy measurements

Attenuation-based transmission X-ray tomographic microscopy experiments are carried out at the 32-ID-C beamline of the Advanced Photon Source at the Argonne National Laboratory at a beam energy of 9.1 keV. For each tomographic scan, 901 projections with an exposure time of 3.5 s (ca. 55 min per scan) are acquired with a 2448 × 2048 pixel detector (73.2 × 61.2 μm field of view), resulting in a voxel size of 29.9 nm. The invar sample holder drastically reduces thermally induced movements during data acquisition and thus suppresses image artifacts.

### Transmission X-ray tomographic microscopy data processing

The projection data are filtered (7 × 7 median filter) and reconstructed with 1000 iterations of the Simultaneous Iterative Reconstruction Technique (SIRT) in the Astra tomography toolbox environment^58^. Data segmentation and the calculation of the gap between the silicon particles and the carbon black-binder domain are performed in MATLAB and are described in Supplementary Notes 5 and 7.

### Scanning electron microscopy sample preparation

The nanoparticle electrode samples are vacuum infiltrated with triphenyl bismuth stained epoxy (Buehler, EpoThin2) to ensure mechanical stability and improve SEM backscatter contrast (see Supplementary Note 2). A total of 1 × 2 mm samples are cut from the infiltrated electrodes and their cross-sections are polished using a Hitachi Broad Ion beam milling system 4000 and sputter-coated with a 2 nm chromium metal layer (Supplementary Note 4).

### Scanning electron microscopy measurement

SEM imaging is carried out on a JEOL scanning electron microscope at an acceleration voltage of 5 kV and a working distance of 8 mm using the compositional backscatter detector. For each sample, a set of five images was acquired with a field of view of 40 × 50 μm and a pixel length of 21 nm.

### Scanning electron microscopy data processing

Data segmentation and distance evaluation between the silicon particles and the carbon black-binder domain are performed in MATLAB (Supplementary Notes 6 and 8). The 2D SEM data are stereographically transformed to allow for comparison with the 3D TXTM data (Supplementary Note 9).

### 1D degradation model

The 1D degradation model presented in Fig. 4 is derived and explained in Supplementary Note 10. All computations are performed in MATLAB.

### 3D renderings

Renderings are assembled using 3D Slicer (open source) and NX10 (Siemens).

### Data availability

The data acquired and analyzed for the study at hand are available from the corresponding author on reasonable request.

## Electronic supplementary material

Supplementary Information
