# Supplementary material for: Quantification and modeling of mechanical degradation in lithium-ion batteries based on nanoscale imaging
Source: Nat Commun. 2018 Jun 14;9:2340. doi: 10.1038/s41467-018-04477-1 (PMC6002379; doi:10.1038/s41467-018-04477-1)
Supplement: Supplementary file 1 — Supplementary Information [file 41467_2018_4477_MOESM1_ESM.pdf]

Supplementary Information

Quantification and modeling of mechanical degradation in lithium-ion batteries based on nanoscale imaging

Müller and Pietsch et al.

SUPPLEMENTARY FIGURES

a

	CB	H	NP	C-Ref.
graphite (wt.%)	70.0	64.1	59.1	85.0
SiOx (wt.%)	15.0	13.7	12.7	0.0
carbon black (wt.%)	5.0	2.3	0.0	5.0
copper nano-particles (wt.%)	0.0	10.8	19.8	0.0
PVDF binder (wt.%)	10.0	9.2	8.4	10.0

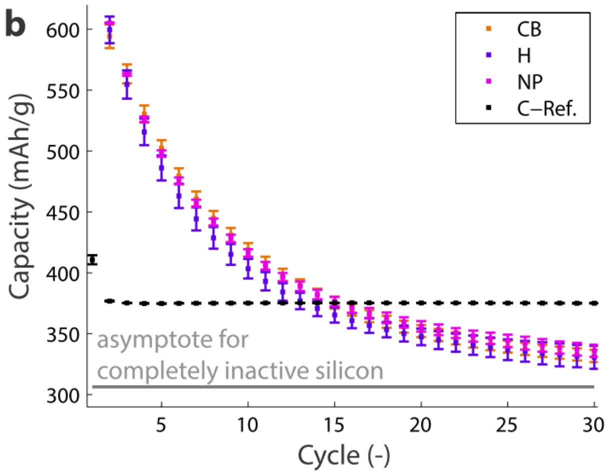

Supplementary Figure 1 | Electrode fabrication and electrochemical cycling. (**a**) Weight fractions of the different constituents in the carbon black (CB), hybrid (H), nanoparticle (NP) and graphite reference (C-Ref.) electrodes. (**b**) Cycling performance of the same electrodes assembled in cells with a lithium counter electrode. Each data point and the associated error bar correspond to the average and the standard deviation of two samples. While the graphite electrodes cycles stable, the specific charge capacity of the silicon-graphite mixed electrodes degrades quickly due to a decreasing amount of electrochemically accessible silicon.

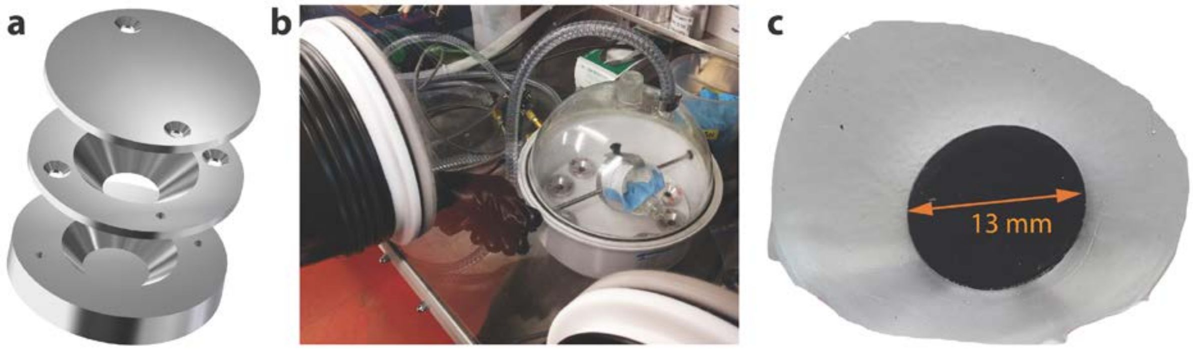

Supplementary Figure 2 | Vacuum infiltration of electrodes. (**a**) Rendering of the custom aluminium holder for microstructure infiltration with epoxy. (**b**) Picture of the vacuum infiltration process in the glovebox. (**c**) Infiltrated electrode disk after removal from the infiltration holder.

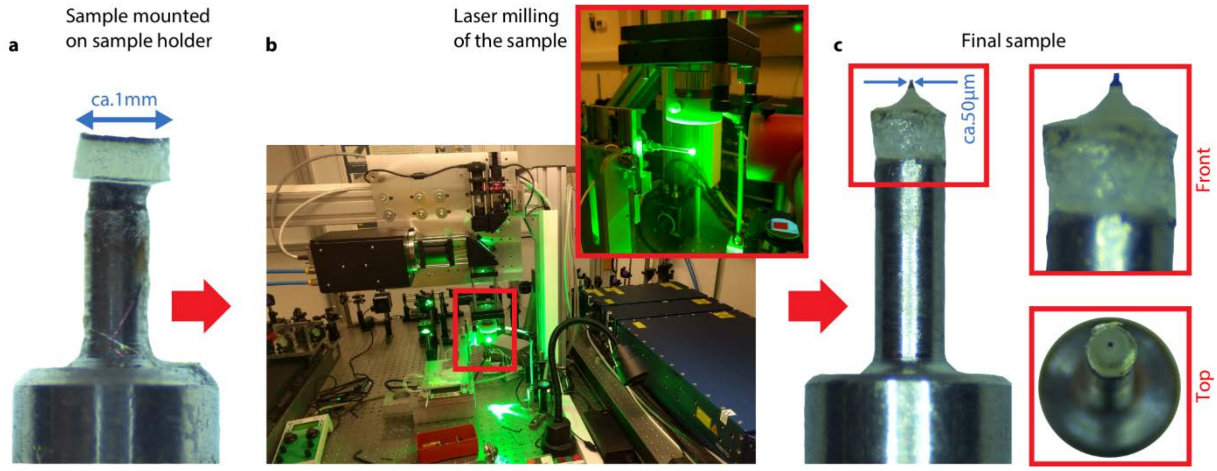

Supplementary Figure 3 | Transmission X-ray tomographic microscopy (TXTM) sample preparation. (**a**) Punched out sample mounted on custom invar sample holder. (**b**) Pictures of the laser milling process. (**c**) Result of the milling process: diameter of the cylindrical electrode is 50 , which is suitable to fit the TXTM field of view.

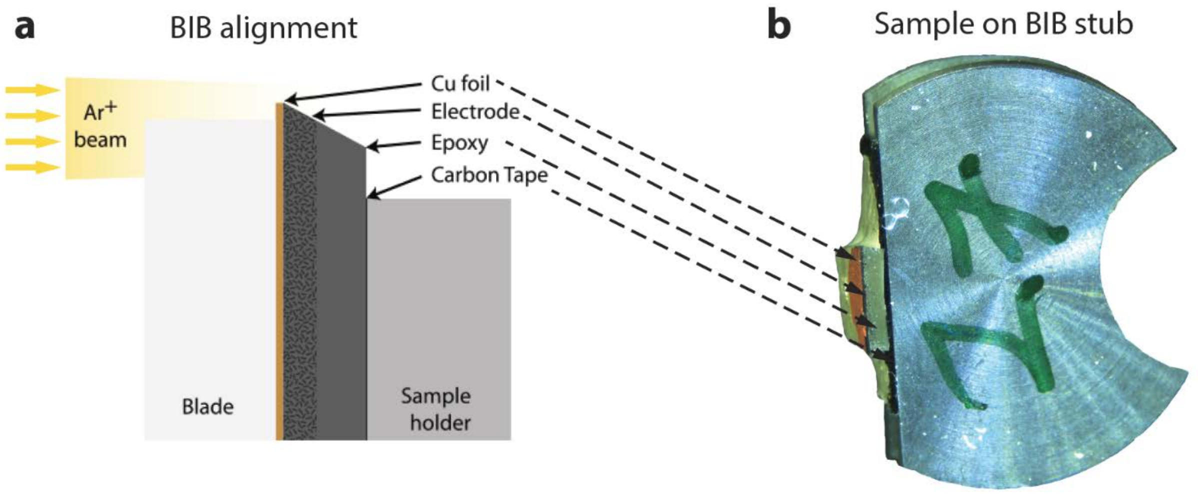

Supplementary Figure 4 | Scanning electron microscopy (SEM) sample preparation. (**a**) Infiltrated electrode sample mounted on a BIB/SEM stub using conductive carbon tape and silver paste. (**b**) Sketch of the BIB alignment.

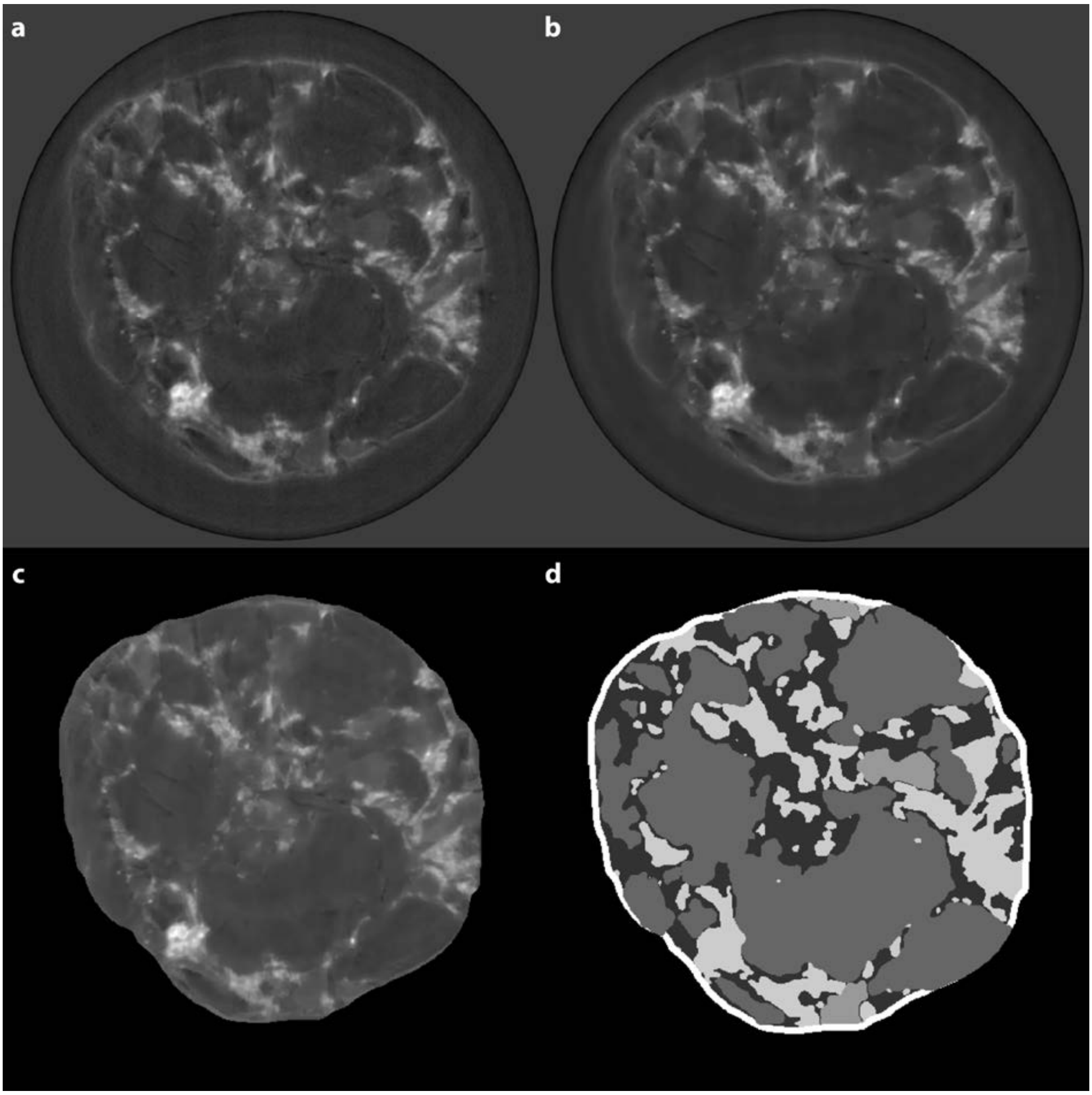

Supplementary Figure 5 | TXTM segmentation. (**a**) Slice of a tomographic raw data set. (**b**) Same slice after application of 20 iterations of a Perona Malik filter. (**c)** Same slice after background removal. (**d**) Segmented sample. From dark to bright the different grey levels represent pore space, graphite, silicon and the carbon black-binder domain, while the white border indicates the zone, where heat from the laser milling has distorted the sample (heat affected zone). This zone is removed for further image processing.

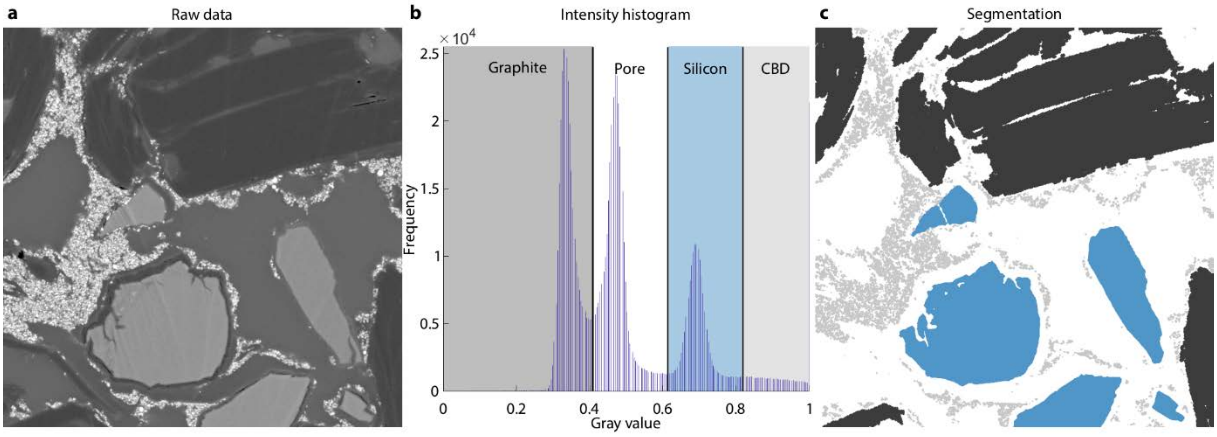

Supplementary Figure 6 | SEM segmentation. (**a**) Crop from a raw image. (**b**) Intensity histogram - the four different phases are clearly distinguishable. (**c**) Final segmentation of image into pore space (white), graphite (black), silicon (blue), and carbon black-binder (grey).

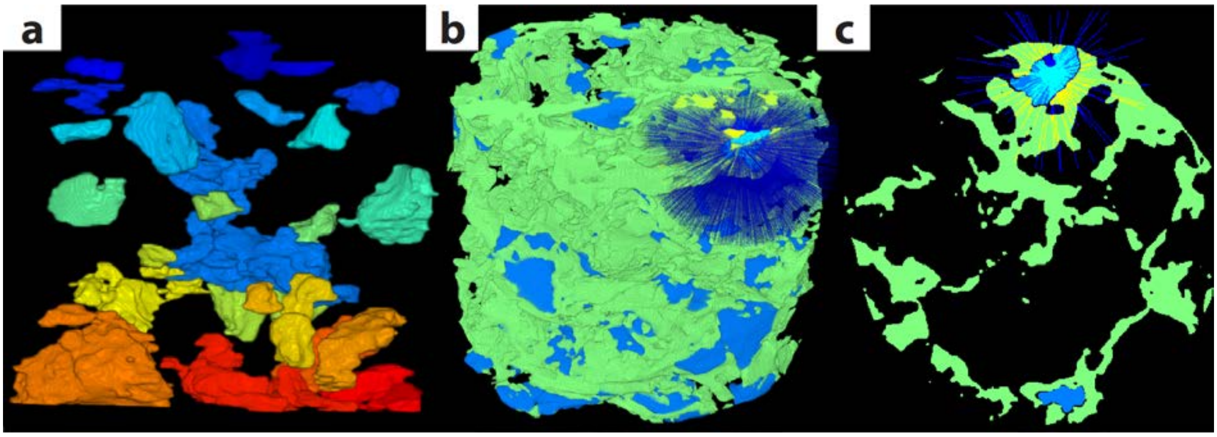

Supplementary Figure 7 | TXTM distance analysis. (**a**) Segmented and labelled silicon particles in a sample. (**b**) Illustration of the silicon particles (blue) embedded in the carbon black-binder domain (green). The virtual rays (dark blue) for the distance analysis are shown for one particle. (**c**) Horizontal cut through the visualization in (b).

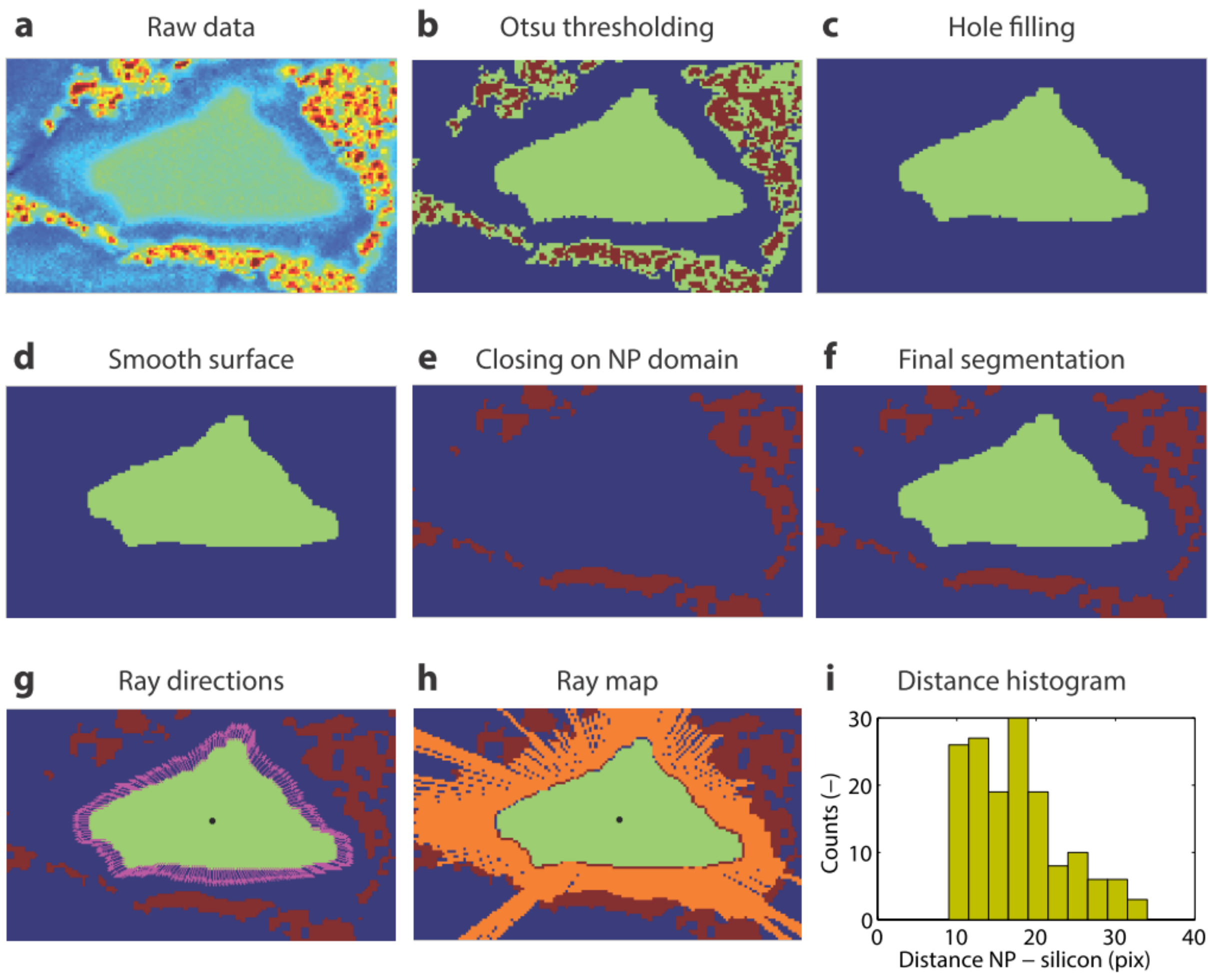

Supplementary Figure 8 | SEM image processing steps. (**a**) Scanning electron microscope raw image data of a silicon particle in the nanoparticle electrode. (**b**) Preliminary trinarization of the data into the silicon phase (green), the nanoparticle phase (red) and the background (blue) using Otsu's automatic thresholding algorithm[7]. (**c**) The silicon particle is identified as the largest connected component and holes are filled. (**d**) Smoothing of the silicon particle surface using morphological operations. (**e**) Closing of the nanoparticle domain. (**f**) Final trinarization of the data. (**g**) Virtual ray directions (magenta) are calculated that point along the connecting lines from the particle centre of mass (black dot) to the respective surface points. (**h**) Virtual rays (orange) start from each particle surface point along the corresponding virtual ray direction and end once they touch any part of the nanoparticle domain. (**i**) Histogram of the silicon surface – nanoparticle domain distances in pixels (1 pixel = 21.3 nm) as measured from the length of the respective rays. Rays that do not hit the nanoparticle domain in a given image are not included in the histogram.

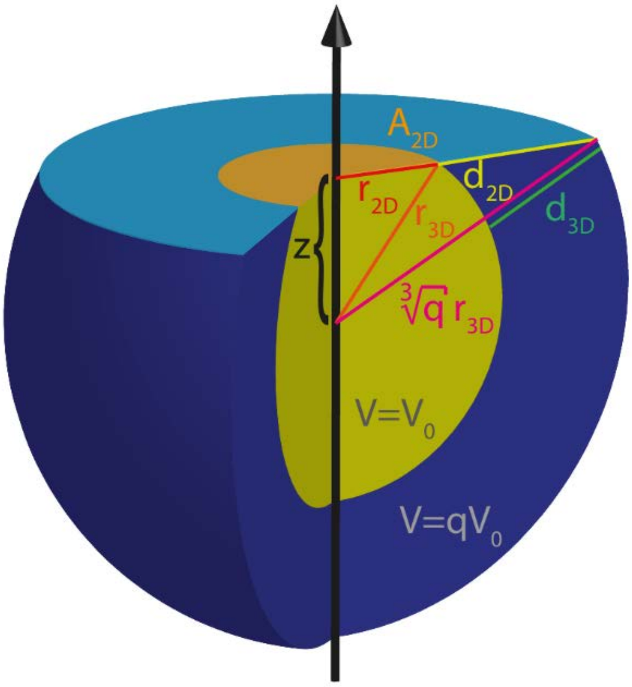

Supplementary Figure 9 | Stereographic conversion. Sketch highlighting the geometric relations of the different 2D and 3D variables as explained in Supplementary Note 9.

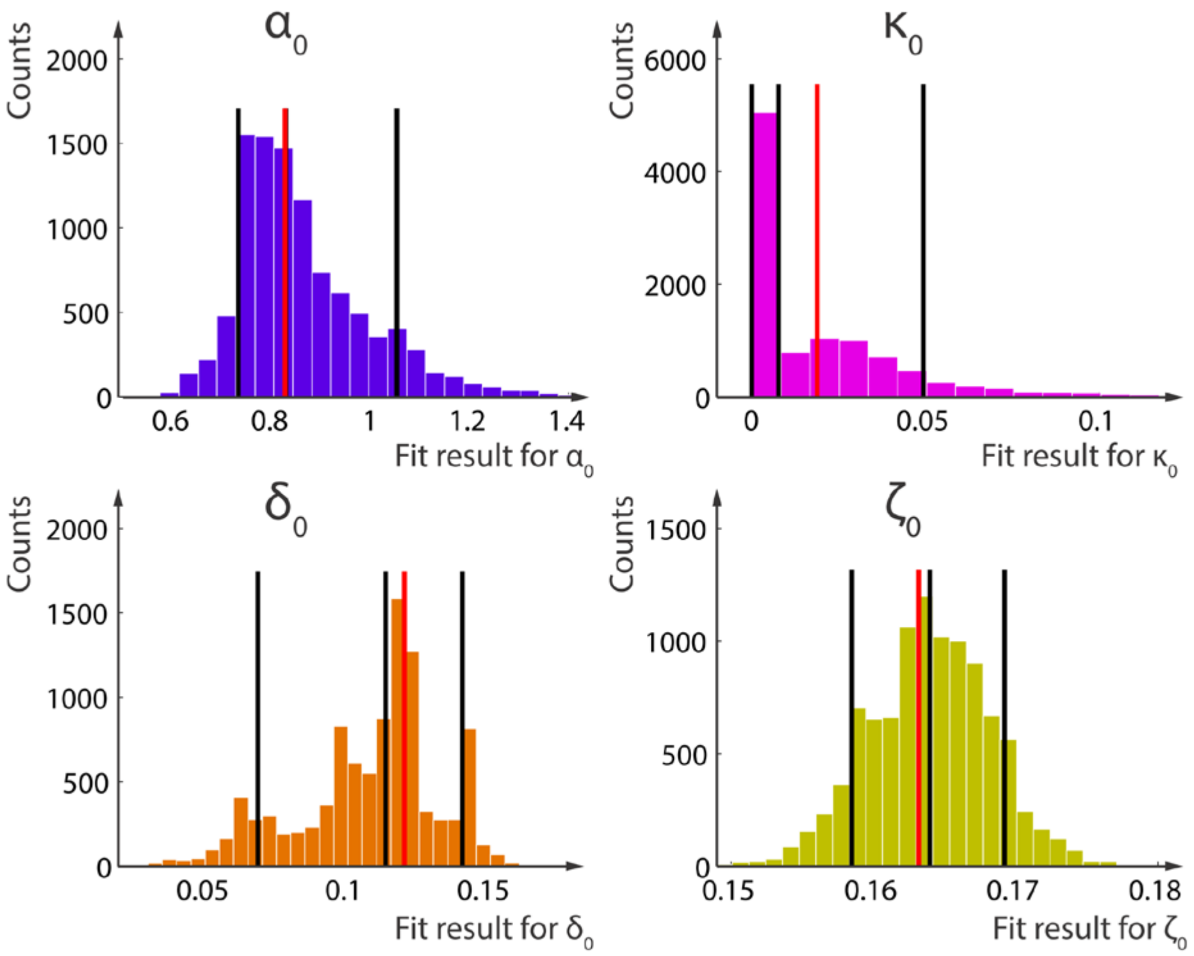

Supplementary Figure 10 | Bootstrapping of the model fitting parameters. Bootstrapping distributions of the α_0, κ_0, δ_0 and ζ_0 fitting parameters of the model. From left to right the vertical black lines in each histogram correspond to the 10th, 50th (median) and 90th percentiles. The red bar corresponds to the fitting values obtained for the actual (i.e. not bootstrapped) data.

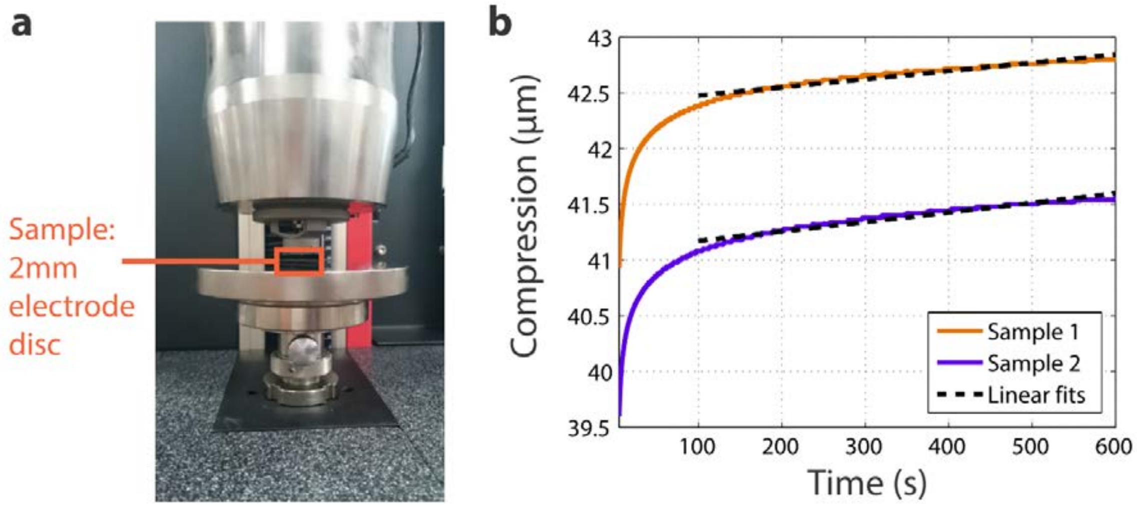

Supplementary Figure 11 | Viscosity measurements. (**a**) Sample stage of the Zwick/Roell Z5.0 machine used to measure stress-strain relations of electrode coatings. (**b**) Absolute compression of two electrode samples as function of time for a constant applied pressure of $100\ \frac{kg}{cm^2}$ ($9.81\ MPa$).

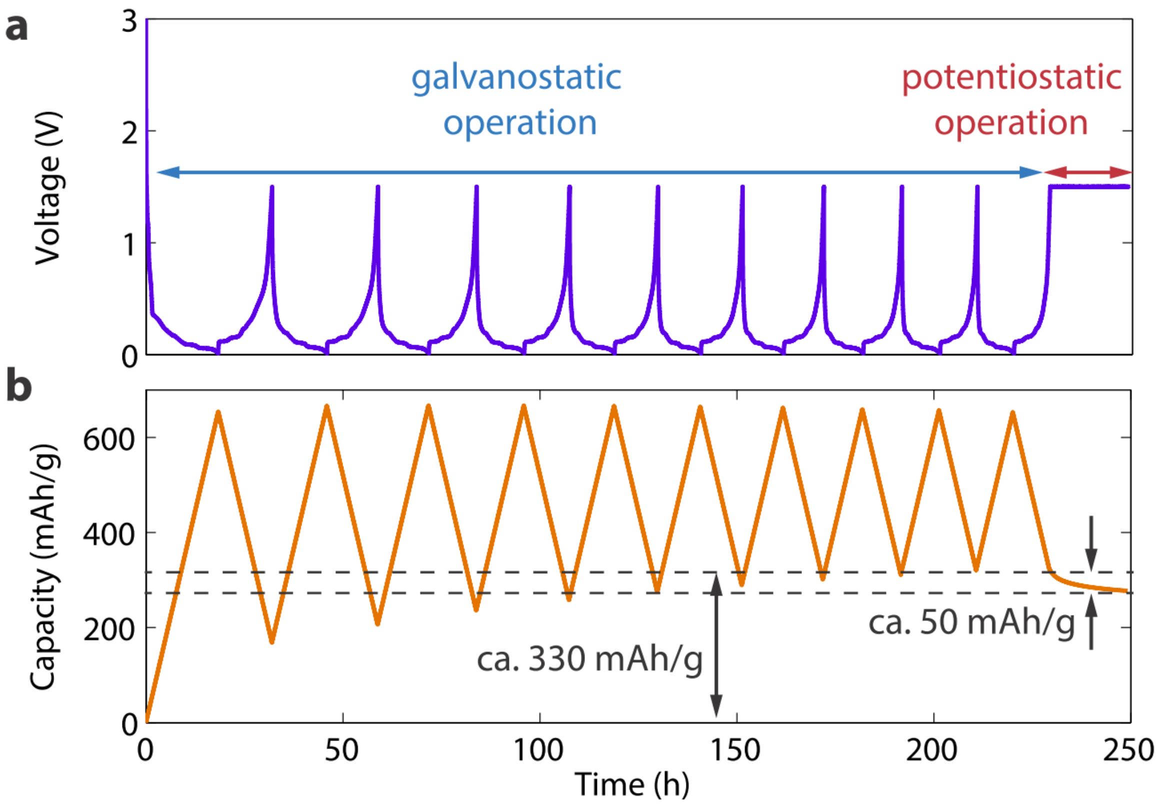

Supplementary Figure 12 | Lithium Trapping (**a**) Voltage and (**b**) cumulative charge in a representative silicon-graphite electrode as a function of operation time. After the electrode has been galvanostatically cycled at a C/20 rate ten times against a lithium metal counter electrode, a 20 h potentiostatic step at 1.5 V has was added. The rather small amount of lithium (ca. 50 $\frac{mAh}{g}$) released in the potentiostatic step is small compared to the overall capacity loss (ca. 330 $\frac{mAh}{g}$), indicating that (kinetic) trapping of lithium in the silicon active materials is not the dominating effect responsible for the loss of cyclable capacity.

SUPPLEMENTARY NOTES

Supplementary Note 1: Fabrication of electrodes and cells, electrochemical cycling

(I) Fabrication of electrodes

Four different silicon-graphite mixed electrodes were fabricated and electrochemically cycled using different graphite, silicon, conductive additive, and binder fractions.

(i) The carbon black electrode (CB) contained 70 wt.% graphite (Hitachi, MagE, ca. $372\ \mathrm{mAh/g}$), 15 wt.% silicon (BASF, $\mathrm{SiO_x}$ with $x \approx 1$, ca. $2275\ \mathrm{mAh/g}$), 5wt.% carbon black conductive additive (Imerys, C65) and 10 wt.% polyvinylidene difluoride (PVDF) binder (Kynar Flex® HSV900).

(ii) For the hybrid electrode (H), 50 vol.% of the carbon black was replaced by carbon coated copper nanoparticles, such that the volume of the conductive additive was conserved (i.e. x g carbon black is replaced by $\frac{x}{2}$ g carbon black and $\frac{x}{2}\frac{\rho_{Cu}}{\rho_{CB}}$ g copper nanoparticles, where $\rho_{Cu} \approx 8.94\ \frac{\mathrm{g}}{\mathrm{cm^3}}$ is the density of copper and $\rho_{CB} \approx 1.9\ \frac{\mathrm{g}}{\mathrm{cm^3}}$ is the approximate density of carbon black). The ratio between the graphite and silicon active materials was kept the same as for the CB electrode.

(iii) For the nanoparticle electrode (NP), all carbon black was volumetrically replaced by carbon coated copper nanoparticles (i.e. x g carbon black was replaced by $x\frac{\rho_{Cu}}{\rho_{CB}}$ g copper nanoparticles). Again, the ratio between the graphite and silicon active materials was kept the same as for the CB electrode.

(iv) For the pure graphite reference electrode (C-Ref.), silicon was replaced by an equal mass of additional graphite.

We considered the silicon-graphite mix as an effective active material, with (active) mass $m = m_{Si} + m_{Gr}$ and effective specific charge capacity $C = \frac{m_{Si}C_{Si}+m_{Gr}C_{Gr}}{m_{Si}+m_{Gr}}$, where m_{Si}, m_{Gr} are the individual masses of silicon and graphite and C_{Si}, C_{Gr} are their specific charge capacities.

Supplementary Figure 1a summarizes the weight fractions for the different electrodes. The fabrication procedure for all electrodes was follows:

(i) Conductive additive and binder (predissolved at 5.2 wt.% in N-Methyl-2-pyrrolidone (NMP)) were iteratively mixed two times in a Thinky planetary mixer at 2000rpm for 2min and sonicated for 2min.

(ii) Addition of silicon was followed by another planetary mixing and sonication step.

(iii) Addition of graphite is followed by another planetary mixing step. The slurry is then mixed in a high shear mixer for 5min. Further NMP is added to optimize the viscosity of the slurry for further processing.

(iv) After a defoaming step in the Thinky mixer (2200 rpm, 2 min) the slurry was coated on a copper foil using a doctor blade with a gap of 150 µm (ca. 80µm after drying).

(v) The electrodes were dried at 80°C under constant nitrogen flow for several hours.

(vi) Disk shaped electrodes with a diameter of 13mm were punched out from the electrode sheets and hydraulically compressed at $6.5 \ \frac{\mathrm{kN}}{\mathrm{cm}^2}$ (thickness after compression ca. $60 \ \mu\mathrm{m}$).

(II) Cell assembly

Electrochemical half cells were assembled in an argon filled glove box, using the 13mm electrodes $250 \ \mu\mathrm{m}$-thick glassfibre separator (Whatman® glass microfiber filter), lithium foil as counter electrode (Alfa Aesar, lithium foil, 99.9 %), and $500 \ \mu\mathrm{l}$ of electrolyte (BASF LP50, 1M $\mathrm{LiPF_6}$ in 50/50 wt.% ethyl-methyl carbonate/ethylene carbonate).

(III) Electrochemical cycling

Cells with the CB, H and NP electrodes were galvanostatically cycled once, three times, and ten times at an effective $C/20$ rate using Biologic MPG2 and VMP3 battery cycling systems. At the end of the last delithiation cycle, a potentiostatic step at 3V was appended to the protocol for 20 h to minimize the amount of lithium remaining in the electrodes. **Supplementary Figure 1b** shows cycling data of the different electrodes over 30 cycles. The CB, H, and NP electrodes perform the same, suggesting that the applied staining technique does not significantly affect the electrochemical behaviour of the electrodes. Conclusions drawn from the imaging experiments of the H and NP electrodes in the main text can therefore be assumed to be applicable to the unstained CB electrode. The control experiment with the pure graphite electrode indicate no significant capacity fade, suggesting that the loss of specific charge capacity in the CB, H, and NP mixed electrodes arises from the loss of active silicon material only. Indeed, after prolonged cycling, all mixed electrodes converge towards the specific charge capacity asymptote that would be expected if silicon was completely inactive (solid gray line).

Supplementary Note 2: Vacuum infiltration of electrodes

All cycled electrodes were vacuum infiltrated with (triphenyl-bismuth (TPB) stained[1,2]) epoxy in order to (i) improve mechanical stability, (ii) provide protection against air and humidity, (iii) provide enhanced image contrast between the different materials, and (iv) facilitate subsequent sample preparation steps. After electrochemical cycling the electrochemical cells were dismantled in an argon filled glovebox and the electrodes were rinsed several times with DMC and dried on a hot plate at 70 °C for 2h prior to infiltration.

For cross-sectional SEM sample preparation the infiltration procedure consists of the following steps:

(i) The electrode is tightly mounted in the custom holder (**Supplementary Figure 2a**) between the lower and the middle part. Holder and sample are then preheated to 95°C in a vacuum oven.

(ii) TPB and epoxy resin (Buehler EpoThin2) is mixed in a weight ratio ≈1:1.6 and heated to 95°C.
(iii) Epoxy hardener is then added, such that the final weight ratio is TPB : resin : hardener = 1 : 1.6 : 0.8 (ca. 30 wt.% TPB).
(iv) The resulting mixture is vortexed and 75µl are cast on the electrode sample.
(v) After 10 min vacuum impregnation, the vacuum oven is vented and the samples are hardened for 1 h at 95°C.

Due to the controlled amount of mixture that was cast on the samples, this procedure results in embedded electrode microstructures with a well-defined overall film thickness of about 500 µm. Care was taken to limit air exposure of the samples to a minimum during the entire procedure. TPB homogeneously dissolves in epoxy[2] and contains heavy bismuth atoms that increase the SEM backscatter signal[3] from the infiltrated pore space regions. The TPB concentration was tuned, such that the grey value of the stained pore space was in the middle between that of graphite and that of silicon, thereby allowing for optimal segmentation of all phases in the SEM images.
For TXTM sample preparation, the infiltration procedure consisted of the following steps:

(i) The electrode was tightly mounted in the custom holder (**Supplementary Figure 2a**) between the lower and the middle part. The samples were placed in a vacuum infiltration gauge, located in an argon-filled glove box (**Supplementary Figure 2b**).
(ii) Epoxy resin and hardener (Buehler EpoThin2) were mixed in a weight ratio 2 : 1, vortexed and moved into the glove box.
(iii) The epoxy mixture was cast on the samples under vacuum (**Supplementary Figure 2b**).
(iv) After venting the vacuum, the top part of the sample holder (**Supplementary Figure 2a**) was mounted, which limited the film thickness of the infiltrated electrode sample to 500 µm.
(v) After 9 h in the glove box the cured samples were removed from the holder (**Supplementary Figure 2c**)

Because no TPB staining and no heating was required for the TXTM sample preparation, vacuum could be applied already during the epoxy pouring step, which results in superior infiltration results compared to the procedure for the SEM samples. Another advantage is that the entire infiltration procedure could be performed in the glove box, such that the interior of the cycled electrodes never came in contact with air.

Supplementary Note 3: TXTM sample preparation
Discs 1 mm in diameter were punched out of the infiltrated electrode and mounted on top of a custom made invar sample holder using superglue (**Supplementary Figure 3a**).
The mounted electrode samples were milled down to a diameter of approximately 50 µm with a Time Bandwidth Duetto solid state laser that generates pulses of a width smaller than 12 ps at a wavelength of 532 nm and an average power of up to 4 W. The laser beam is deflected to the desired position using a micro scanning system that is based on a galvanometer-driven scan head and passes through an optical arrangement to be finally focused on a focal spot of 10 µm (see **Supplementary Figure 3b** for a picture). This laser line setup available at inspire AG

(Zurich) is described in detail by Gottman et al.[4]. **Supplementary Figure 3c** shows the samples from different perspectives after the milling process. Lasers have already been used elsewhere to micro-machine samples for subsequent X-ray nano-tomography measurements[5].

Supplementary Note 4: SEM sample preparation
The infiltrated electrodes were cut to 1mm x 2mm pieces and mounted on a stub suitable for broad ion beam (BIB) milling using carbon tape and conductive silver paste (**Supplementary Figure 4a**). **Supplementary Figure 4b** illustrates the BIB principle: a beam of argon ions polishes the cross-section over a metal blade. Milling and polishing was performed for 60-90min using a Hitachi Broad Ion Beam 4000 system at 4kV acceleration voltage and 1.5kV beam emission voltage. After the polishing process the sample was immediately coated with a 5nm thin chromium layer prior to SEM imaging.

Supplementary Note 5: TXTM segmentation
First, the three dimensional 16bit image stacks (**Supplementary Figure 5a**) were normalized to the range between 0 and 1. Then, 20 iterations of the anisotropic diffusion Perona Malik filter[6] were applied (**Supplementary Figure 5b**). Since not the entire field of view was filled out by the sample, peripheral regions were cut off such that only the actual sample data is left (**Supplementary Figure 5c**). This is done by making use of the fact that the grey values at the sample border are brighter than of the empty surroundings. The actual segmentation algorithm differentiates between four phases. First, the carbon black-binder domain phase (bright green) is identified using three dimensional hysteresis thresholding. Second, to tag the silicon phase, normal grey value based thresholding was used and the carbon black-binder was subtracted. The result was eroded and then dilated again to its original edges to get rid of small residues. The infilling of intra-particle pores and five three dimensional smoothing iterations with a Gaussian kernel conclude the segmentation of the silicon phase. Third, the identification of the graphite phase was done similarly, except for a lower threshold value. Finally, all remaining pixels were attributed to the pore space. The result is shown in **Supplementary Figure 5d**. All computations were performed in MATLAB.
The laser milling of the sample can lead to burnt regions at the very edge of the sample, resulting in brighter grey values. To avoid confusion with the also bright silicon or carbon black-binder domain phase a fifth phase, the heat affected zone, is introduced. This region was not taken into account for the further analysis.

Supplementary Note 6: SEM segmentation
In a first step, the intensities of the SEM raw images (**Supplementary Figure 6a**) were normalized to the range between 0 and 1. Based on the histogram of the grey values (**Supplementary Figure 6b**), a multi-threshold segmentation procedure was implemented. Three threshold values were chosen at the respective points where the absolute gradient of the histogram is minimal between two neighbouring peaks, resulting in four phases: the very bright carbon black-binder, the light grey silicon particles, the darker pore space and the almost black graphite particle phase. The silicon and graphite phase were further treated with morphological operations. An erosion was followed by a dilation to the original edges. In addition, only particles containing more than 1000 pixels are taken into account in order to get rid of residues. Finally, intra-particle porosities are infilled. The result is shown in **Supplementary Figure 6c**. All computations were performed in MATLAB.

Supplementary Note 7: TXTM distance analysis

To evaluate the three dimensional distance between the surface of each silicon particle and the surrounding carbon black-binder domain, the single particles in the silicon phase have to be identified (**Supplementary Figure 7a**). The procedure for each particle was the same. From the particle's center of mass a large number of rays was sent in uniformly distributed directions (**Supplementary Figure 7b and c**). Every ray was evaluated with respect to its period of residence in the silicon phase, the pore space, or the carbon black-binder domain. The eventual distance from a silicon particle to the carbon black-binder domain results from the averaged distances along all rays. The corresponding effective radius of the silicon particles was defined as $r_{\mathrm{eff}} = \sqrt[3]{\frac{3V}{4\pi}}$, where V is the volume of the particle.

Supplementary Note 8: SEM distance analysis

To determine the average gap size between the surface of a silicon particle and the carbon black-binder domain, we first cropped all silicon particles individually (**Supplementary Figure 8a**). Otsu thresholding[7] allowed for a first estimate of the three phases: pore space (blue), the silicon particles (green), and the carbon black-binder domain (red) (**Supplementary Figure 8b**). We then isolated the silicon particle by picking the largest connected component, filled in enclosed domains in the particle (**Supplementary Figure 8c**), and slightly smoothed the surface (**Supplementary Figure 8d**). To compensate for the parts around the carbon black-binder domain that have been falsely identified as silicon, the nanoparticle domain is isolated and slightly expanded using a morphological closing operation (**Supplementary Figure 8e**). This results in the final segmentation (**Supplementary Figure 8f**). Subsequently the centre of mass of the silicon particle is determined and from each point on the silicon particle surface virtual rays are sent along the direction that connects the centre of mass with the corresponding surface point (**Supplementary Figure 8g and h**). For each ray that hits the carbon black-binder domain, the corresponding silicon surface to carbon black-binder domain distance is measured and added to a histogram (**Supplementary Figure 8i**). The average of these distances defines the gap that is associated with that silicon particle. Its effective radius is defined as $r_{\mathrm{eff}} = \sqrt{A/\pi}$, where A is the area of the particle.

Supplementary Note 9: Stereo conversion

In order to quantitatively compare the 2D SEM data with the 3D tomography data, we performed a stereo conversion for the respective carbon black binder domain – silicon surface distances and the effective radii of the silicon particles. For the derivation of the conversion factors, we assumed that all silicon particles are spheres that expand and contract uniformly. We further assume that 2D cross-sections through a silicon particle occur with the same probability at different positions z. For the definition of the different variables used in the equations below, we refer to the sketch in **Supplementary Figure 9**.

For the conversion between the expectation value of the 2D effective radius $< r_{2D} >$ and the 3D effective radius r_{3D}, we find:

$$< r_{2D} > = \frac{1}{r_{3D}} \int_0^{r_{3D}} \sqrt{{r_{3D}}^2 - z^2} dz = \frac{\pi}{4} r_{3D}$$

$$\frac{< r_{2D} >}{r_{3D}} = \frac{\pi}{4} \cong 0.785$$

In analogy the conversion between the expectation-value of the 2D distances $< d_{2D} >$ and the 3D distance d_{3D} is given by:

$$\frac{< d_{2D} >}{d_{3D}} = \frac{\frac{1}{r_{3D}}\int_0^{r_{3D}} dz\, d_{2D}(z)}{\left(q^{\frac{1}{3}}-1\right) r_{3D}} = \frac{\frac{q^{\frac{2}{3}}}{2}\operatorname{asin}\left(q^{-\frac{1}{3}}\right) + \frac{q^{\frac{2}{3}}}{4}\sin\left(2\operatorname{asin}\left(q^{-\frac{1}{3}}\right)\right) - \frac{\pi}{4}}{q^{\frac{1}{3}}-1}$$

As described in **Supplementary Note 9**, q is the factor by which the silicon particles expand upon full lithiation. Based on the specific charge capacity of the material used (SiO_x with $x \approx 1$, ca. $2275\ \mathrm{mAh/g}$), we estimate $q \approx 2.6$ (160 % volume expansion), yielding a ratio:

$$\frac{< d_{2D} >}{d_{3D}} \cong 1.217$$

Scaling the respective axis in the plots showing the distance as a function of the effective radius allows to convert the 2D SEM data and the 3D tomography data into one another.

Supplementary Note 10: 1D degradation model

In the following, we derive the mathematical components of the 1D degradation model.

(I) 1D volumetric changes of the silicon upon (de)lithiation

We assume the silicon particles are spheres with a volume $V = \frac{4}{3}\pi r^3$, where r is the effective radius of the silicon particle.

A silicon particle that volumetrically expands by a factor q upon lithiation and contracts by the same amount upon delithiation thus changes its radius by an absolute amount:

$$d_{exp} = \left(\frac{3qV}{4\pi}\right)^{\frac{1}{3}} - \left(\frac{3V}{4\pi}\right)^{\frac{1}{3}} = \left(\frac{3V}{4\pi}\right)^{\frac{1}{3}}\left(q^{\frac{1}{3}}-1\right) = r\left(q^{\frac{1}{3}}-1\right)$$

(II) Solid electrolyte interface growth

The thickness of the solid electrolyte interface (SEI) on silicon is assumed to grow linearly in time[8] during the lithiation processes with some proportionality factor δ:

$$d_{SEI}(i) \propto t = \delta \sum_{m=1}^{i} T(m) = \delta \sum_{m=1}^{i} \frac{C(m)}{I}$$

where $T(m)$ is the lithiation time in cycle m, I is the galvanostatic current and $C(m)$ is the measured lithiation capacity of the electrode in the electrochemical cycle m. Because the current I is constant in our galvanostatic experiments, defining $c(i) = \frac{C(i)}{C(i=0)}$ as capacity fraction in cycle i allows one to rewrite the expression to:

$$d_{SEI}(i) \coloneqq \delta_0 \sum_{m=1}^{i} c(m)$$

with $\delta_0 = \frac{\delta\, C(i=0)}{I} = \delta T_0$ being the effective growth rate of the solid electrolyte interface.

(III) Maxwell model for an electrode with effective viscoelastic properties

As discussed in the main text, the Maxwell model[9] is one of the simplest equivalent circuits that can be used to describe a material with viscoelastic properties. It consists of a damper element in series with a spring element. For a damper element the stress σ_D is proportional to the rate of the strain ϵ_D via $\sigma_D = \eta \frac{d\epsilon_D}{dt}$ while, for the spring element, the stress σ_S is directly

proportional to strain ϵ_S via $\sigma_S = E\epsilon_S$. Here, η and E are the material's effective viscosity and elastic modulus, respectively.
Since the elements are connected in series, the elongations are additive ($\epsilon \coloneqq \epsilon_{tot} = \epsilon_D + \epsilon_S$), while the stresses must be equal ($\sigma \coloneqq \sigma_{tot} = \sigma_D = \sigma_S$). This results in the differential equation:

$$\frac{d}{dt}\epsilon = \frac{1}{\eta}\sigma + \frac{1}{E}\frac{d}{dt}\sigma.$$

However, since we model an absolute displacement between the surface of the silicon particles and the carbon-black-binder domain, we rewrite the differential equation to:

$$\frac{d}{dt}d = \frac{1}{\bar{\eta}}\sigma + \frac{1}{\bar{E}}\frac{d}{dt}\sigma,$$

The absolute distance $d = \epsilon d_{ref}$ is linked to a reference distance d_{ref} modifying the viscosity and the elastic modulus to $\bar{\eta} = \frac{\eta}{d_{ref}}$ and $\bar{E} = \frac{E}{d_{ref}}$, respectively. The reference distance, d_{ref} can be thought of as some effective distance over which a compression propagates into the material. Precise knowledge of d_{ref} is not needed for the model to be applied.
For simplicity, we assume that the displacement responds linearly to silicon (de)lithiation ($d \propto t$). Let $T(i)$ be the (de)lithiation time in cycle i. During lithiation the elongation $d(t,i,r)$ as a function of time in cycle i for a silicon particle with radius r will be given by:

$$d(t,i,r) = \left[d_{exp}(r) + d_{SEI}(i) - d_{SEI}(i-1)\right]\frac{t}{T(i)} + d_{SEI}(i-1)$$

Correspondingly, during delithiation:

$$d(t,i,r) = -d_{exp}(r)\frac{t}{T(i)} + d_{exp}(r) + d_{SEI}(i)$$

The elongations are counted relative to the surface of delithiated silicon particle in the pristine electrode. The solutions of the differential equation during lithiation and delithiation are thus given by

$$\sigma(t,i,r) = \gamma e^{-\frac{\bar{E}}{\bar{\eta}}t} + \left[d_{exp}(r) + d_{SEI}(i) - d_{SEI}(i-1)\right]\frac{\bar{\eta}}{T(i)}$$

and

$$\sigma(t,i,r) = \gamma e^{-\frac{\bar{E}}{\bar{\eta}}t} - d_{exp}(r)\frac{\bar{\eta}}{T(i)}$$

respectively, with γ being an integration constant.
At the beginning of a lithiation cycle i, when the SEI (re)touches the surrounding electrode material, the stress is zero: $\sigma(t=0) = 0$. From this condition, the integration constant can be calculated, which yields:

$$\sigma(t,i,r) = \left[d_{exp}(r) + d_{SEI}(i) - d_{SEI}(i-1)\right]\frac{\bar{\eta}}{T(i)}\left(1 - e^{-\frac{\bar{E}}{\bar{\eta}}t}\right)$$

At the end of a lithiation process of an electrochemical cycle i, the electrode material around the silicon particle is compressed by the thickness of the additional SEI grown in this cycle plus the amount by which the electrode material relaxed in the previous cycle $d_{relax}(i-1,r) + d_{SEI}(i) - d_{SEI}(i-1)$. Divided by the rate of elongation during lithiation $\frac{d}{dt}d(t,i,r) = \left[d_{exp}(r) + d_{SEI}(i) - d_{SEI}(i-1)\right]\frac{1}{T(i)}$, we obtain the time τ in cycle i during which the surrounding electrode material is in touch with the expanding silicon particle and compressed by it:

$$\tau(i,r) = \frac{d_{relax}(i-1,r) + d_{SEI}(i) - d_{SEI}(i-1)}{d_{exp}(r) + d_{SEI}(i) - d_{SEI}(i-1)}T(i)$$

We thus find a corresponding tension of

$$\sigma(i,r) = \left[d_{exp}(r) + d_{SEI}(i) - d_{SEI}(i-1)\right]\frac{\bar{\eta}}{T(i)}\left(1 - e^{-\frac{\bar{E}}{\bar{\eta}}\tau(i,r)}\right)$$

at the end of lithiation cycle i.

At the beginning of the delithiation process in cycle i the initial condition $\sigma(t=0) = \sigma(i,r)$ allows determination of γ, such that

$$\sigma(t,i,r) = \left(\left[d_{exp}(r) + d_{SEI}(i) - d_{SEI}(i-1)\right]\frac{\bar{\eta}}{T(i)}\left(1 - e^{-\frac{\bar{E}}{\bar{\eta}}\tau(i,r)}\right) + d_{exp}(r)\frac{\bar{\eta}}{T(i)}\right)e^{-\frac{\bar{E}}{\bar{\eta}}t} - d_{exp}(r)\frac{\bar{\eta}}{T(i)}.$$

During the delithiation process, the electrode material follows the retreating silicon particle until detachment occurs when a critical adhesion value $\sigma = x$ is exceeded (or no detachment occurs if the particle is fully delithiated, before the critical value $\sigma = x$ is reached).

With $\sigma(t=0) = \sigma(i,r)$ as initial condition, detachment or full delithiation happens after a time

$$T = \min\left[\frac{\bar{\eta}}{\bar{E}}\ln\left[\frac{1 + \left(1 + \frac{d_{SEI}(i) - d_{SEI}(i-1)}{d_{exp}(r)}\right)\left(1 - e^{-\frac{\bar{E}}{\bar{\eta}}\tau(i,r)}\right)}{1 + \frac{xT(i)}{\bar{\eta}d_{exp}(r)}}\right], T(i)\right]$$

which yields the corresponding distance by which the electrode relaxes in each cycle i:

$$d_{relax}(i,r) = \frac{d_{exp}(r)}{T(i)}T.$$

We introduce $\alpha(i) = \frac{T_i\bar{E}}{\bar{\eta}} = \frac{T_0E}{\eta}c(i) := \alpha_0 c(i)$ and $\kappa(i) = -\frac{T_i x}{\bar{\eta}} = -\frac{xT_0 d_{ref}}{\eta}c(i) =: \kappa_0 c(i)$ to find:

$$d_{relax}(i,r) = d_{exp}(r)\min\left[\frac{1}{\alpha(i)}\ln\left[\frac{1 + \left(1 + \frac{d_{SEI}(i) - d_{SEI}(i-1)}{d_{exp}(r)}\right)\left(1 - \exp\left[-\alpha(i)\frac{d_{relax}(i-1,r) + d_{SEI}(i) - d_{SEI}(i-1)}{d_{exp}(r) + d_{SEI}(i) - d_{SEI}(i-1)}\right]\right)}{1 - \frac{\kappa(i)}{d_{exp}(r)}}\right], 1\right]$$

(*)

This formula recursively defines the relaxation distance.

α_0 represents the effective material properties in terms of the elastic modulus and the viscosity.

κ_0 represents the critical adhesion at which the carbon-black-binder domain detaches from the silicon particle upon delithiation.

(IV) Calculating the silicon surface to nanoparticle front distance

The position of nanoparticle front at the end of lithiation step i is given by (relative to surface of delithiated silicon):

$$p_{lith}(i,r) = d_{exp}(r) + d_{SEI}(i)$$

Correspondingly, the position of the nanoparticle front at the end of delithiation step I is given by (relative to surface of delithiated silicon):

$$p_{delith}(i,r) = d_{exp}(r) + d_{SEI}(i) - d_{relax}(i,r)$$

With the initial conditions:

$$d_{relax}(i=0,r)=d_{exp}(r)$$
$$d_{SEI}(i=0)=0$$

equation (*) can be iterated to obtain $d_{relax}(i,r)$ explicitly.
The distance between the surface of a silicon particle and the nanoparticle domain is then given as a function of the cycle number i and the effective particle radius r by:

$$d(i,r)=p_{delith}(i,r)=d_{exp}(r)+d_{SEI}(i)-d_{relax}(i,r)$$

(V) Drop-out probability

In order to relate the calculated distances between the carbon-black-binder domain and the silicon particle surfaces $d(i,r)$ to the electrochemistry, we introduce a drop-out probability function $p(d(i,r))$, which describes the probability of a silicon particle with radius r to drop out in cycle i, depending on the corresponding distance $d(i,r)$. For this model, we assume, that the drop out probability is only a function of the distances $d(i,r)$.
Considering only silicon particles with radius r, the fraction of particles that are still active after i cycles is then given by the product:

$$s(i,r)=\begin{cases}1 & \text{if } \ i=0\\ \prod_{m=1}^{i}[1-p(d(m,r))] & \text{if } \ i>0\end{cases}$$

The capacity fraction $c(i)$ still available after i cycles can then be calculated from:

$$c(i)=\frac{\sum_k s(i,r_k)\,c_{\mathrm{Si}}(r_k)}{\sum_k\, s(i=0,r_k)\,c_{\mathrm{Si}}(r_k)}=\frac{\int_0^\infty dr\,\mathrm{PSD}(r)\,s(i,r)\,r^3}{\int_0^\infty dr\,\mathrm{PSD}(r)\,r^3}$$

where k is an index running over all particles with effective radius r_k in the electrode, $c_{Si}(r)=\frac{4}{3}\pi r^3 c_{V,Si}$ represents the charge capacity of a spherical silicon particle with effective radius r ($c_{V,Si}$ is the volumetric charge capacity of silicon), and $\mathrm{PSD}(r)$ is the silicon particle size distribution.
Thus, if the probability distribution $p(d)$ and the parameters α_0, κ_0, δ_0 are known, the above implicit definition of $c(i)$ can be self-consistently solved to predict electrochemical behavior. (note that s(i,r) also depends on $c(i)$, because $d(i,r)$ does).
We estimated the particle size distribution $\mathrm{PSD}(r)$ from the SEM data by fitting a generalized extreme value distribution in the sense of least squares.
The functional shape of the dropout probability function can be defined in many different ways. For simplicity and to avoid overfitting, we postulated a linear relation that predicts zero dropout probability for a distance $d=0$ (i.e. when the carbon-black-binder domain is still attached to the silicon particle):

$$p(d)=\zeta_0 d,$$

where ζ_0 is the only fitting parameter of the distribution.

(VI) Calculate statistical expectation values

The derived equation for $d(i,r)$ describes the silicon surface to nanoparticle distance for a particle with effective radius r that has been cycled for i electrochemical cycles. To compare our

results to the experimental image data, the expectation value of the distribution of distances $< d(i,r) >$ has to be calculated.

As discussed in the main text and presented in Figure 1c, the amount of silicon contributing to the effective specific charge capacity fades with time. We assume that a particle either fully contributes to the measured capacity (i.e. it is electrically connected to the network) or it does not contribute at all. I.e. a particle never contributes partially to the capacity. We also assume that when a particle is active until cycle i and then turns inactive, the corresponding nanoparticle to silicon distance remains at the calculated value and thus remains unchanged by the mechanical activity of the remaining electrode parts in further cycles.

With $s(i,r)$ being the fraction of active silicon particles with effective radius r after i cycles, we find:

$$< d(i,r) > = \left(\sum_{m=1}^{i} (s(m-1,r) - s(m,r))\, d(m-1,r)\right) + s(i,r)d(i,r)$$

Note that $\left(\sum_{m=1}^{i} s(m-1,r) - s(m,r)\right) + s(i,r) = s(i=0,r) = 1$ for normalization purposes.

The corresponding standard deviation in the observed distance distribution arising from different particles stopping (de)lithiation at different cycles can be calculated from:

$$\sigma(i,r) = \sqrt{\left(\sum_{m=1}^{i} (s(m-1,r) - s(m,r))\, (d(m-1,r) - < d(i,r) >)^2\right) + s(i,r)(d(i,r) - < d(i,r) >)^2}$$

(VII) Solving the model

The entire model is implemented in MATLAB based on custom code. Using an initial set of parameters α_0, κ_0, δ_0, ζ_0 we first update α_0, κ_0, δ_0 using a fit (using the MATLAB routine lsqnonlin(), 1-norm, trust-region-reflective-algorithm) by comparing the model prediction $< d(i,r) >$ to the experimental distance data. In this process the initial guess for ζ_0 remains unchanged. In a second step, we update ζ_0, by comparing the model's implicit definition of the capacity fraction $c(i)$ with the experimental electrochemical data (using again lsqnonlin()). In this step the updated parameters α_0, κ_0, δ_0 from the first step are kept constant. These two steps are repeated to iteratively update α_0, κ_0, δ_0, ζ_0, until the model converges to a self-consistent solution with relative tolerance 1E-6.

Once the optimal parameters are found, individual parameters can be varied and the model can be once again solved for $c(i)$ to predict and model electrochemical behavior.

(VIII) Estimation of the uncertainty ranges for α_0, κ_0, δ_0 and ζ_0 using bootstrapping

In order to estimate the robustness of our fit and 80 % confidence intervals for the four parameters α_0, κ_0, δ_0 and ζ_0, we perform bootstrapping[10].

The measured image data used as input for the original fit consists of a vector with 412 triplet entries (cycles i, radii r and distances d for each of the 412 analyzed silicon particles). We then generate 10.000 bootstrapping samples, where each sample has the same size as the original data (412 (i, r, d) data triplets), but consists out of a random combination of the original (i, r, d) data triplets (taking the same triplet multiple times in a sample is allowed).

For each of these bootstrapping samples the model is solved according to the description in (VII). The resulting distributions of the optimized α_0, κ_0, δ_0 and ζ_0 values are shown in **Supplementary Figure 10.**

For each distribution we calculate the 10th, 50th (median) and 90th percentiles, which allows us to establish 80 % confidence intervals around the values that we obtain from fitting the original data (red lines in **Supplementary Figure 10**): $\alpha_0 = 0.83^{+0.23}_{-0.09}$, $\kappa_0 = 0.02^{+0.03}_{-0.02}$ μm, $\delta_0 = 0.12^{+0.02}_{-0.05}$ μm and $\zeta_0 = 0.163^{+0.006}_{-0.005}$ μm^{-1}.
The relative uncertainty ranges are thus < 7 % for ζ_0, < 39 % for α_0 and < 61 % for δ_0. While these ranges are reasonable and show that the extracted parameters are robust against changes in the recorded data, κ_0 can be found anywhere in the range [0 μm, 0.05 μm] (uncertainty > 260 %, note that κ_0 is constrained to be positive by fitting routine). As can be seen from the corresponding histogram in **Supplementary Figure 10,** it is even very likely that $\kappa_0 = 0$ μm. Thus, while we are able to provide an upper bound for κ_0 ($\kappa_0 < 0.05$ μm) our model does not allow for a precise quantification of the adhesion from the available image data. However, this large uncertainty in κ_0 does not significantly affect the estimates for the other parameters: setting the constraint $\kappa_0 = 0$ μm in the fitting routing yields $\alpha_0 = 0.79$, $\delta_0 = 0.1$ μm and $\zeta_0 = 0.164$ μm^{-1}, which is very close to the above reported results. We can therefore keep κ_0 in the model, which allows us to investigate its effect on the electrochemical degradation rate. Note that except in case of κ_0, the 50th percentiles (medians) of the α_0, δ_0 and ζ_0 parameters are very close to the values we calculate from our original dataset (red lines in **Supplementary Figure 10**), implying that our dataset represents the statistics very well.

Supplementary Note 11: Viscosity measurements

For a constant stress σ, the differential equation of the Maxwell model simplifies to:

$$\frac{d}{dt}\epsilon = \frac{1}{\eta}\sigma$$

The viscosity η can thus be obtained from measuring the rate of compression of the electrode material at a constant applied pressure. This experiment (see **Supplementary Figure 11a**) was performed for two different samples using a Roell/Zwick Z5.0 machine at a pressure of $100\ \frac{\text{kg}}{\text{cm}^2}$ (9.81 MPa). A linear fit of the data is used to extract an order of magnitude estimate of the viscosity:
$\eta \approx 1000$ GPas (**Supplementary Figure 11b**).

SUPPLEMENTARY REFERENCES

1. Zhu, W., Liu, F. & He, J. Synthesis of Radio-Opaque Methacrylate Monomer and its Application in Visible Light-Curable Dental Resin. *Adv. Polym. Technol.* **0,** 1–7 (2016).

2. Salamone, J. in *Polymeric materials encyclopedia: Q-S* 7346–7350 (1996).

3. Goldstein, J. I. *et al. Scanning Electron Microscopy and X-Ray Microanalysis*. *Microscopy* (Springer US, 1992). doi:10.1007/978-1-4613-0491-3

4. Gottmann, J., Hermans, M. & Ortmann, J. Digital Photonic Production of Micro Structures in Glass by In-Volume Selective Laser-Induced Etching using a High Speed Micro Scanner. *Phys. Procedia* **39,** 534–541 (2012).

5. Bailey, J. J. *et al.* Laser-preparation of geometrically optimised samples for X-ray nano-CT. *J. Microsc.* **267,** 384–396 (2017).

6. Ebner, M. Designing Better Batteries: Visualization and Quantification of Microstructure and Degradation Mechanisms in Lithium Ion Battery Electrodes. (ETH Zuerich, 2013).

7. Otsu, N. A threshold selection method from gray-level histograms. *IEEE Trans. Syst. Man. Cybern.* **9,** 62–66 (1979).

8. Pinson, M. B. & Bazant, M. Z. Theory of SEI Formation in Rechargeable Batteries: Capacity Fade, Accelerated Aging and Lifetime Prediction. *J. Electrochem. Soc.* **160,** A243–A250 (2012).

9. Gutierrez-Lemini, D. *Engineering Viscoelasticity*. *Engineering Viscoelasticity* (Springer US, 2014). doi:10.1007/978-1-4614-8139-3

10. Efron, B. & Tibshirani, R. J. *An Introduction to the Bootstrap*. (Chapman & Hall/CRC, 1994).
